# Interaction of Late Apoptotic and Necrotic Cells with Vitronectin

**DOI:** 10.1371/journal.pone.0019243

**Published:** 2011-05-04

**Authors:** Ondrej Stepanek, Tomas Brdicka, Pavla Angelisova, Ondrej Horvath, Jiri Spicka, Petr Stockbauer, Petr Man, Vaclav Horejsi

**Affiliations:** 1 Institute of Molecular Genetics, Academy of Sciences of the Czech Republic, Praha, Czech Republic; 2 Institute of Hematology and Blood Transfusion, Prague, Czech Republic; 3 Institute of Microbiology, Academy of Sciences of the Czech Republic, Praha, Czech Republic; French National Centre for Scientific Research, France

## Abstract

**Background:**

Vitronectin is an abundant plasma glycoprotein identified also as a part of extracellular matrix. Vitronectin is substantially enriched at sites of injured, fibrosing, inflamed, and tumor tissues where it is believed to be involved in wound healing and tissue remodeling. Little is known about the mechanism of vitronectin localization into the damaged tissues.

**Methodology/Principal Findings:**

2E12 antibody has been described to bind a subset of late apoptotic cells. Using immunoisolation followed by mass spectrometry, we identified the antigen recognized by 2E12 antibody as vitronectin. Based on flow cytometry, we described that vitronectin binds to the late apoptotic and necrotic cells in cell cultures in vitro as well as in murine thymus and spleen in vivo. Confocal microscopy revealed that vitronectin binds to an intracellular cytoplasmic structure after the membrane rupture.

**Conclusions/Significance:**

We propose that vitronectin could serve as a marker of membrane disruption in necrosis and apoptosis for flow cytometry analysis. Moreover, we suggest that vitronectin binding to dead cells may represent one of the mechanisms of vitronectin incorporation into the injured tissues.

## Introduction

Apoptosis and necrosis represent two fundamental types of cell death. While necrosis is usually viewed as a more or less passive cell rupture caused by excessive exogenous damage, apoptosis is an active process consisting of highly coordinated molecular events leading to a sequence of morphological changes and is accompanied by modifications of the cellular surface. The cell loses its surface anti-phagocytic “don't-eat-me” signals (mediated mostly by CD31 and CD47 glycoproteins) and exposes ligands designating the cell for phagocytosis (e.g. phosphatidylserine) [1], [2]. Moreover, several extracellular molecules bind to the apoptotic cells (e.g. MFG-E8, TSP-1, complement factors) facilitating phagocytosis [1]–[3]. Importantly, the early apoptotic cells preserve their plasma membrane integrity to retain the potentially harmful cellular contents inside. If not successfully taken up by phagocytes, apoptotic cells proceed to the phase of late apoptosis (termed also secondary necrosis) when the plasma membrane becomes permeable for small molecules (e.g. propidium iodide (PI)) and subsequently also for macromolecules (proteins) [4]. The leakage of intracellular molecules during secondary necrosis provokes an inflammatory response, explaining why defective apoptotic cell clearance is associated with autoimmune diseases [3].

Reagents specifically recognizing the cells at particular stages of apoptosis can be useful research and diagnostic tools. A monoclonal antibody 2E12 has been reported to recognize a subset of apoptotic cells in culture [5], [6]. However, the precise identity of this subset as well as the molecule recognized by this antibody have remained unknown. Here we show that the 2E12 antibody recognizes bovine serum protein vitronectin (originating from cell culture medium) bound to the late apoptotic cells.

Vitronectin is a major plasma glycoprotein produced mainly in the liver where it is released into the circulation [7], [8]. It is also a part of extracellular matrix, substantially enriched at sites of injured, fibrosing, inflamed, and cancer tissue [8]–[12].

Vitronectin was initially described as an inhibitor of complement terminal pathway and a regulator of blood homeostasis [13]–[15]. In addition, it contributes to tissue remodeling and healing by regulation of proteolysis, cell adhesion, migration, and survival in the injured tissue [10], [15]–[22]. Moreover, vitronectin probably enhances migration of leukocytes into the stressed tissue [10], [19], [23]. On the other hand, vitronectin also stimulates tumor invasiveness and contributes to the development of chronic tissue injuries [15], [24], [25]. Known binding partners mediating cell interactions with vitronectin-containing tissues include integrins (α_V_β_3_, α_V_β_5_, α_V_β_1_, α_V_β_6_, α_V_β_8_, and αIIbβ_3_) and the urokinase receptor. In contrast, the mechanism of the transport and deposition of vitronectin in the stressed tissues remains still incompletely understood [8], [15].

Here we bring evidence that vitronectin binds to an intracellular component of cells in the latest stage of apoptosis and of necrotic cells in vitro as well as in vivo, which could represent an important mechanism facilitating vitronectin incorporation into the sites of tissue injury.

## Materials and Methods

### 1. Antibodies and proteins

Antibodies to the following antigens were used: human vitronectin (VN58-1, mouse, Abcam, Cambridge, MA, USA), human albumin (AL-01, mouse, Exbio, Vestec, Czech Republic), human cytokeratin-Alexa Fluor 488 (pan-reactive, Exbio), human vimentin-Dy-547 (Exbio), bovine vitronectin (rabbit, Acris Antibodies, Herford, Germany), mouse vitronectin (347317, rat, R&D Systems, Minneapolis, MN, USA), mouse Ig-HRP (goat, Bio-Rad, Hercules, CA, USA), rabbit Ig-HRP (goat, Bio-Rad), mouse Ig-Alexa Fluor 488 (goat, Invitrogene, Carlsbad, CA, USA), mouse Ig-Alexa Fluor 647 (goat, Invitrogen), and rat Ig-Allophycocyanin (APC) (goat, BD Bioscience, Franklin Lakes, NJ, USA). The following isotype matched controls were used: Rat IgG2a (eBioscience, San Diego, CA, USA) and anti-HLA-DR (mouse IgG1, MEM-12, in house). Following proteins and peptides were used: human vitronectin (Technoclone, Dorking, UK), biotinylated human vitronectin (Cell Sciences, Sharon, MA, USA), bovine vitronectin (Sigma-Aldrich, St. Louis, MO, USA), RGD peptide (Sigma-Aldrich), streptavidin-Alexa Fluor 488 (Invitrogene), Annexin-V-FITC, Annexin-V-Dy647 (both Apronex, Prague, Czech Republic). Mouse monoclonal antibody 2E12 (IgG1) was described previously [5].

### 2. Cell culture

Human cell lines Jurkat (American Type Culture Collection, Manassas, VA, USA), HeLa (provided by D. Stanek, IMG, Prague, Czech Republic), JCaM2.5 (A. Weiss, University of California, San Francisco, CA, USA), Ramos, HL-60 (both American Type Culture Collection), chicken cell line DT40 (J. Wienands, University of Göttingen, Germany), rabbit cell line 240E1 (K. Knight, Loyola University Chicago, Maywood, IL, USA), mouse cell lines SP2/0 (American Type Culture Collection), P815 (H. Stockinger, Medical University of Vienna, Austria) were cultivated in RPMI 1640 medium containing 10% fetal bovine serum (FBS), 2 mM glutamine, 20 µg/ml gentamycin, 50 µg/ml streptomycin, and 10^4^ U/ml penicillin at 37°C in 5% CO_2_.

A green fluorescent protein (GFP) expressing Jurkat cell line (provided by M. Hrdinka, IMG, Prague) was prepared by electroporation (250 V, 950 µF) of pZRD (15 µg) vector [26] in RMPI (300 µl) using a GenePulser electroporator (Bio-Rad Laboratories, Hercules, CA, USA) followed by zeocin selection (200 µg/ml, Invitrogen).

Apoptosis was induced by TRAIL ligand (300 ng/ml, Apronex) or camptothecin (1 µM, Sigma-Aldrich) for the indicated time period. Inhibition of apoptosis was performed with pan-caspase inhibitor Z-VAD-FMK (10 µM, Enzo Life Sciences, Inc., Farmingdale, NY, USA). Necrosis was induced by incubation at 65°C for 40 minutes or by hydrogen peroxide (2 mM) in combination with Z-VAD-FMK (10 µM).

### 3. Flow cytometry and confocal microscopy

Viable, apoptotic, or necrotic Jurkat cells were stained with a primary antibody followed by a corresponding secondary antibody (both in PBS/1% BSA, on ice, 30 min). Alternatively, cells were incubated with biotinylated vitronectin (3.6 µg/ml) followed by streptavidin-Alexa Fluor 488 (both in PBS/1% BSA, on ice, 30 min). Stages of cell death were examined by staining with Annexin-V, PI (1 µM, Sigma-Aldrich), and/or Hoechst 34580 (2 µg/ml, Invitrogen) (Annexin binding buffer or PBS/1% BSA, on ice, 30 min). For DNA content analysis, the cells were incubated with Hoechst 34580 (5 µg/ml, 37°C, 20 min). For microscopy, cells were transferred into Lab-Tek chambers (Thermo Fisher Scientific, Waltham, USA) in Annexin binding buffer.

HeLa cells were fixed (PBS/4% formaldehyde), permeabilized, and blocked (PBS/5% BSA/0.3% Triton-X 100). The cells were incubated with human serum at 37°C followed by staining with primary and secondary antibodies at room temperature. Serum and antibodies were diluted in PBS/1% BSA/0.3% Triton-X 100. For microscopy, the cells were stained with Hoechst 34580 (2 µg/ml) for 5 minutes.

Spleens and thymi were collected from healthy 8–9 weeks old C57Bl/6j mice (IMG Animal Facility). Single-cell thymocyte or splenocyte suspensions were prepared followed by erythrocyte lysis in ACK buffer. Approximately 2×10^6^ cells were stained with anti-vitronectin or isotype matched control antibodies followed by incubation with APC-conjugated secondary antibody (PBS/20% goat serum, on ice, 30 min). Finally, the cells were stained with Annexin-V-FITC, PI, and Hoechst 34580 (Annexin binding buffer, on ice, 30 min). Mice used as the source of splenocytes and thymocytes were kept under the conditions required by national guidelines for the use of experimental mice and their use was approved by the Animal Welfare Commission of the Institute of Molecular Genetics, Academy of Sciences of the Czech Republic, permit No. 46817/2007 valid for 2007–2011.

Flow cytometry samples were analyzed on LSRII or FACScalibur apparatus (BD Biosciences). Data were analyzed using FlowJo software (TreeStar, San Carlos, CA, USA). Images were acquired with a Leica SP5 confocal microscope using a 100× objective lens (Leica Microsystems, Mannheim, Germany). Data were analyzed using LAS AF 2.00 software (Leica).

### 4. Quantification of GFP release to the medium

4×10^6^ wild type or GFP-expressing Jurkat cells were transferred to fresh RPMI/10% FBS at high cell density (2.5×10^6^ cells/ml) and apoptosis was induced by camptothecin. At indicated time points, 150 µl of the suspension was taken, centrifuged, and the cell free supernatant was kept in 37°C. After the collection of all samples, fluorescence intensity (excitation 475 nm, emission 510 nm) of each sample (100 µl) was measured by InfiniteM200 microplate reader (Tecan Group Ldt., Mannedorf, Switzerland). The GFP fluorescence in medium was obtained by subtraction of the background fluorescence of the respective samples from GFP-negative Jurkat cultures.

### 5. Immunoisolation

2E12 or isotype matched control antibodies were covalently bound to CNBr-activated Sepharose (Sigma-Aldrich) according to the manufacturer's instructions. Immunoaffinity chromatography was performed at 4°C on minicolumns containing 40 µl of the immunosorbent. FBS (100 µl) was applied at the top of the minicolumn followed by PBS wash (400 µl). The adsorbed proteins were eluted with 80 µl of 0.1 M triethanolamine (pH 11.5). Subsequently, pH of the eluted fraction was adjusted with 10 µl 1 M Tris/HCl (pH 8.2). Finally, the material was mixed 1∶1 with 2× concentrated Laemmli sample buffer (non-reducing or reducing with final 0.5% dithiothreitol) and subjected to SDS-PAGE followed by Coomassie Brilliant Blue R-250 (Bio-Rad) staining or immunoblotting.

### 6. Mass spectrometry analysis

Preparation of the sample and analysis by an ion trap mass spectrometer (LCQ^DECA^, ThermoElectron, Waltham, MA, USA) was performed as described previously [27]. The instrument was set to acquire a full MS scan between 350–1800 m/z followed by MS/MS scan of the most intense ion from the preceding scan. The MS/MS data were searched against NCBI non-redundant database with SEQUEST software (Thermo Fisher Scientific) as described previously [28].

### 7. Binding of vitronectin to red blood cells

RBCs were isolated from a blood of a healthy donor using Histopaque-1119 (Sigma-Aldrich) gradient. RBCs (10% v/v in PBS/0.5 mM MgCl_2_/1 mM CaCl_2_) were biotinylated with sulfo-NHS-LC-biotin (300 µM, Pierce, Rockford, IL, USA) on ice for 1 hour. RBCs were washed twice and incubated in the presence of streptavidin (40 µg/ml, Jackson ImmunoResearch, West Grove, PA, USA) on ice for 30 minutes. RBCs were washed twice and incubated with biotinylated human vitronectin (72 µg/ml) on ice for 30 minutes. The blood sample was obtained from one of the authors of this study (O.S.) based on his written consent. The Commission for Ethics and Work with Recombinant DNA and Human Materials of the Institute of Molecular Genetics, Academy of Sciences of the Czech Republic, exempted this study from review because it considered the written informed consent ethically unproblematic.

## Results

### 1. 2E12 antibody specifically binds late apoptotic cells cultured in the presence of bovine serum

**Figure 1:**
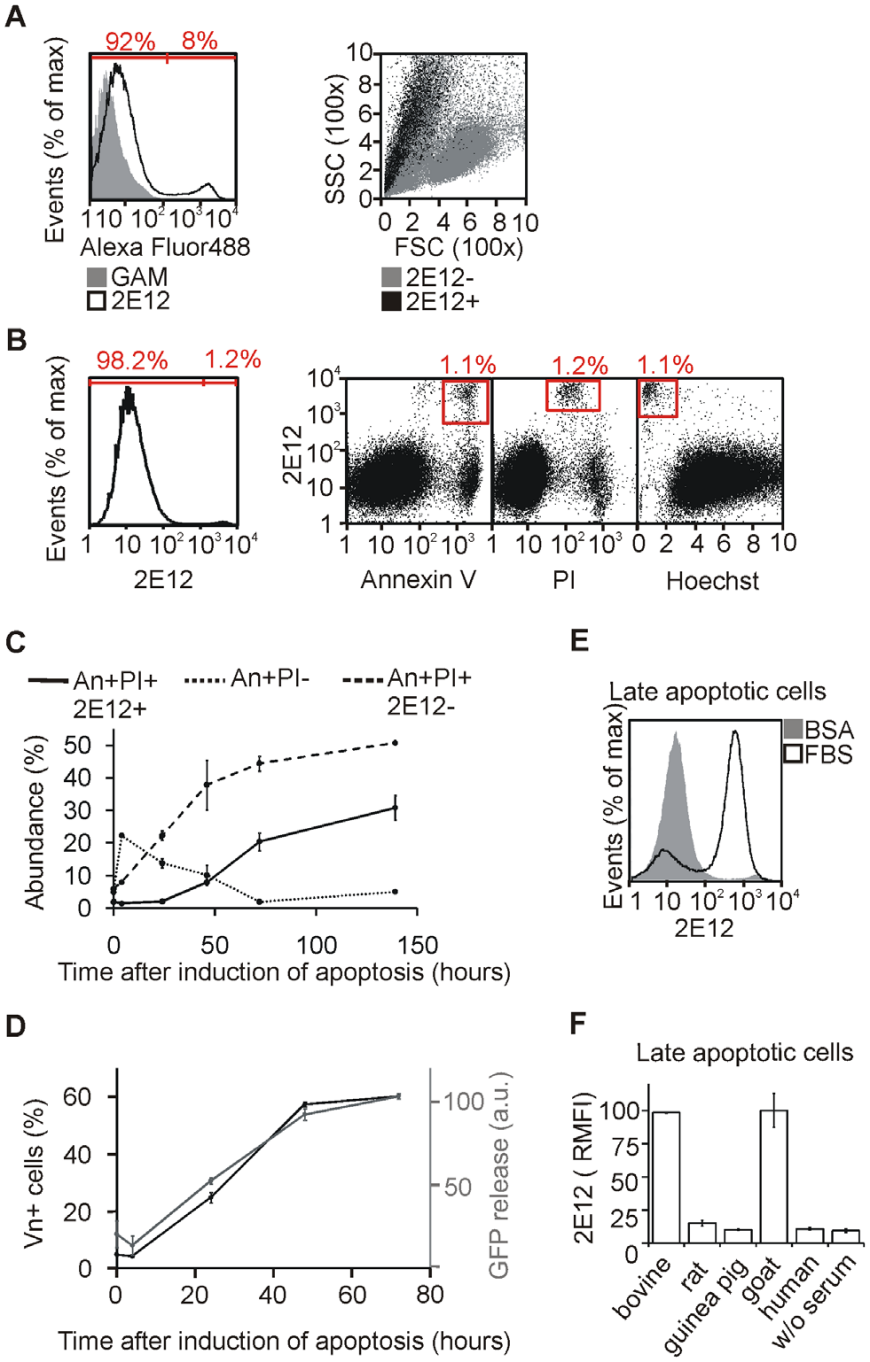
2E12 antibody recognizes a serum component bound to late apoptotic cells. (A) Jurkat cells were stained with 2E12+GAM-Alexa Fluor 488 (black line) antibodies or GAM-Alexa Fluor 488 only (grey filled histogram) and analyzed by flow cytometry (left panel). Forward scatter (FSC) vs. side scatter (SSC) plot shows 2E12 negative (gray) and 2E12 positive (black) Jurkat cells (right panel). The cell culture was grown to density about 2×10^6^ cells/ml. (B) Jurkat cells were stained with Annexin-V, PI, Hoechst 34580, and 2E12 + GAM-Alexa Fluor 647 antibodies and analyzed by flow cytometry. (C) Apoptosis was induced in Jurkat cells by camptothecin. The cells were examined for Annexin-V-FITC, PI, and 2E12 + GAM-Alexa Fluor 647 staining by flow cytometry at indicated time points. Mean ± SD, n = 3. (D) Wild type or intracellular GFP expressing Jurkat cells were transferred to fresh medium prior to apoptosis induction with camptothecin at high cell density (2.5×10^6^/ml). At indicated time points, 150 µl of the culture was taken up. The cells were stained with 2E12 antibody followed by flow cytometry analysis while the cell-free supernatant was collected. The release of GFP to medium in all samples was measured by fluorescence plate reader at the end of the experiment. The values represent net GFP fluorescence after the subtraction of the background fluorescence intensity of the medium from wild type Jurkat cells. Mean ± SD, n = 3. (E) Apoptosis was induced in Jurkat cells by TRAIL in serum-free medium (containing 1% BSA). After 72 hours, one half of the cells were incubated in 50% FBS for 1 hour. Finally, both 50% FBS-treated and untreated cells were stained with 2E12 + GAM-Alexa Fluor 488 antibodies and analyzed by flow cytometry. (F) Cells were prepared as in (E) except that the apoptotic cells were incubated with 50% bovine, rat, guinea pig, goat, or human sera or left untreated prior to 2E12 staining and analysis. Mean ± SD, n = 3. RMFI, relative mean fluorescence intensity.

Monoclonal antibody 2E12 recognized a subpopulation of Jurkat T cells in culture, as revealed by flow cytometry (Fig. 1A). The frequency of 2E12-positive cells varied, depending on the viability of the particular culture. Overgrown cultures (approx. 2×10^6^ cells/ml) contained more cells stained with 2E12 than cultures in optimal growth conditions (<10^6^ cells/ml) (Fig. 1A–B). The 2E12-positive cells exhibited features of cell death as indicated by forward and side scatter analysis and Annexin-V staining (Fig. 1A–B). In contrast to previously published data [5], these cells were stained with PI, indicating that the plasma membrane was not intact (Fig. 1B). However, cells, that were most intensively stained with PI, were 2E12-negative. The intermediate PI signal of the 2E12-positive cells can be explained by low DNA content as shown by Hoechst 34580 staining (Fig. 1B).

We induced apoptosis in Jurkat cells and monitored them using Annexin-V, PI, and 2E12 staining over time. We distinguished three populations of apoptotic cells: Annexin-V^+^/PI^−^, Annexin-V^+^/PI^+^/2E12^−^, and Annexin-V^+^/PI^+^/2E12^+^ (Fig. 1C). The Annexin-V^+^/PI^−^ population that peaked early after apoptosis induction and diminished over time, was identified as early apoptotic cells. Secondary necrotic cells defined as Annexin-V^+^/PI^+^ appeared later. Interestingly, the increase of Annexin-V^+^/PI^+^/2E12^+^ cells was observed after 48 hours, suggesting that the 2E12-positive cells represent the latest stage of apoptosis progression.

We performed a comparison between the 2E12-positivity and intracellular protein release after apoptosis induction over time. Jurkat cell line producing cytosolic GFP was subjected to apoptosis induction with camptothecin at high cell density. The kinetics of the GFP release to the medium was strikingly similar to the increase of the percentage of the 2E12-positive cells over time (Fig. 1D). This observation suggests that the cells stained with 2E12 are the source of GFP released to the medium. The 2E12 antibody obviously recognizes cells with severely damaged plasma membrane, permeable for large molecules like proteins.

We tested six cell lines of different origin (human, chicken, rabbit, and mouse) cultured in the presence of fetal bovine serum (FBS) for 2E12 positivity. In all cases a hypodiploid 2E12-positive population was detected (Fig. S1). This led us to hypothesize that the 2E12 antibody might recognize a bovine serum component specifically bound to late apoptotic cells. Thus, we prepared late apoptotic Jurkat cells in serum-free medium followed by a short incubation in FBS prior to 2E12 staining. The serum-treated cells, but not cells held in serum-free conditions, became 2E12-positive (Fig. 1E), which pointed to the serum origin of the molecule recognized by the 2E12 antibody. Extension of this assay to sera from different species revealed that short incubation in bovine or goat but not in rat, guinea pig, or human sera resulted in recognition of the late apoptotic cells by the 2E12 antibody (Fig. 1F).

### 2. 2E12 antibody recognizes bovine vitronectin

**Figure 2:**
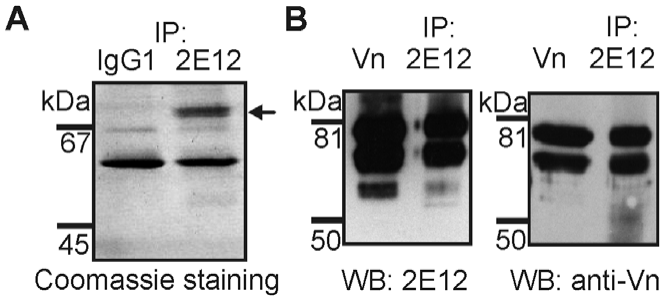
2E12 antibody recognizes bovine serum vitronectin. (A) Immunosorbents made of 2E12 or isotype-matched control antibodies were used for immunoisolation from bovine serum of the antigen recognized by the 2E12 antibody. The immunoisolated material was separated by non-reducing SDS-PAGE followed by Coomassie staining. The band isolated specifically by 2E12 is marked by black arrow. (B) Purified vitronectin standard or the 2E12 immunoprecipitate were immunoblotted (reducing conditions) and stained with 2E12 (left membrane) or commercial rabbit antibody to bovine vitronectin (right membrane). Vn, vitronectin.

To identify the bovine serum component recognized by the 2E12 antibody, we subjected the serum to immunoisolation on immobilized 2E12 antibody. Separation of the immunoprecipitated material by SDS-PAGE under non-reducing conditions followed by Coomassie Blue protein staining detected a single major protein (ca 70–80 kDa) that was immunoisolated using the 2E12 antibody but not an isotype matched control antibody (Fig. 2A). The stained zone was cut from the gel, trypsin digested, and analyzed by tandem mass spectrometry. Bovine vitronectin (accession number 78045497) was the only protein identified in the sample. The final protein coverage was 39% with 15 peptides spread over the whole protein sequence (AA 38 to 476). To confirm that 2E12 antibody is specific for bovine vitronectin, we immunoblotted purified vitronectin standard side by side with the material immunoisolated by means of the 2E12 antibody. Both 2E12 antibody and a commercial polyclonal antibody to bovine vitronectin stained the immunoisolated material as well as the vitronectin standard (Fig. 2B). Furthermore, when unseparated FBS was subjected to Western blotting, 2E12 antibody detected only a doublet corresponding by m.w. to vitronectin and not any other of the multiple bovine serum components (not shown). Thus, it can be safely concluded that the 2E12 antibody recognizes bovine vitronectin and that the material immunoisolated from FBS by means of the 2E12 antibody contains vitronectin (Fig. 2B). The presence of the two bands observed in the Western blots is caused by reduction of the sample with dithiothreitol that reveals the previously described single-chain (75 kDa) and two-chain (65+10 kDa) vitronectin forms [7].

### 3. Vitronectin binds late apoptotic cells directly

**Figure 3:**
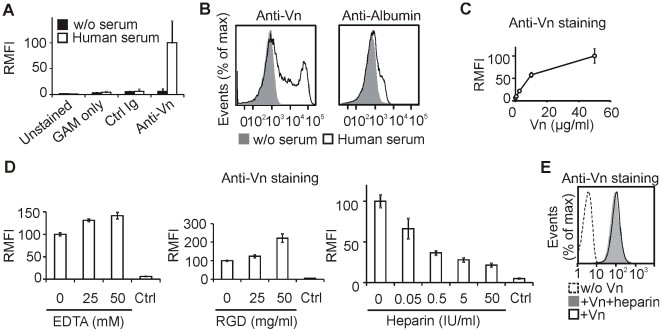
Heparin inhibits the direct interaction between vitronectin and late apoptotic cells. Apoptosis was induced in Jurkat cells by TRAIL in serum-free medium (containing 1% BSA) and left for 48 or 72 hours. (A) The late apoptotic cells were incubated in 30% human serum or serum-free medium for 1 hour. The cells were stained with anti-vitronectin or isotype matched control antibodies followed by GAM-Alexa Fluor 488 antibody staining and analyzed by flow cytometry. Unstained cells and cells stained only with GAM-Alexa Fluor 488 served as additional controls. Mean ± SD, n = 3. (B) The late apoptotic cells were incubated in 50% human serum or serum-free medium for 1 hour and stained with anti-vitronectin or anti-albumin and GAM-Alexa Fluor 647 antibodies for flow cytometry analysis. Mean ± SD, n = 3. (C) The late apoptotic cells were incubated in serum-free medium supplemented with various concentrations of human purified vitronectin for 1 hour. The cells were stained with anti-vitronectin and GAM-Alexa Fluor 488 antibodies and analyzed by flow cytometry. Mean ± SD, n = 3. (D) The late apoptotic cells were incubated in the presence of 30% human serum and various concentrations of EDTA, RGD, or heparin. The cells were stained with anti-vitronectin GAM-Alexa Fluor 488 antibodies and analyzed by flow cytometry. Cells that were not pre-incubated in human serum served as negative controls. Mean ± SD, n = 3. (E) Biotinylated human vitronectin was bound onto RBCs using biotin-streptavidin-biotin sandwich approach. RBCs were incubated in the presence or absence of heparin (50 IU/ml) in serum-free RPMI at 37°C for 1 hour and subsequently stained with anti-vitronectin antibody for flow cytometry analysis. RBCs without bound vitronectin were used as a negative control. One representative experiment of 2 is shown.

After we identified that bovine serum vitronectin binds to late apoptotic cells, we decided to study the interaction in more detail. We switched to a purely human model system to avoid any potential complications resulting from interspecies incompatibilities at the molecular level and repeated the vitronectin binding assay using Jurkat cells, human serum, and antibody to human vitronectin. The late apoptotic cells were again recognized by anti-vitronectin antibody only after incubation in human serum (Fig. 3A). The percentage of human vitronectin-positive cells increased after apoptosis induction when cultivated in human serum-containing medium (Fig. S2) and followed similar kinetics to 2E12-positive cells in RMPI/10% FBS (Fig. 1C). To test whether binding to late apoptotic cells is a specific feature of vitronectin, we performed staining of late apoptotic cells with antibodies to human vitronectin and human albumin. Although albumin is 300 times more abundant than vitronectin in plasma [29] and the anti-albumin antibody produced a much stronger signal than the anti-vitronectin antibody in ELISA assay on human serum (not shown), the signal produced by anti-albumin antibody on the serum-incubated late apoptotic cells was negligible compared to the anti-vitronectin antibody (Fig. 3B). This indicated the specificity of the vitronectin binding. Given the multiplicity of known vitronectin-binding partners in plasma [7], it was unclear whether vitronectin binds the late apoptotic cells directly or uses another serum component as a molecular bridge. We incubated the late apoptotic cells in serum-free medium with various concentrations of purified vitronectin. Vitronectin bound to the cells in a dose dependent manner indicating a direct interaction between vitronectin and the late apoptotic cells (Fig. 3C).

Live cells are able to interact with vitronectin via several integrin receptors. However, high concentrations of EDTA or the integrin-blocking peptide (RGD) did not prevent binding of serum vitronectin to late apoptotic cells, excluding the possibility that the interaction was mediated by integrins (Fig. 3D). Surprisingly, both EDTA and the RGD peptide even enhanced the signal intensity after anti-vitronectin antibody staining. On the other hand, heparin, a well established vitronectin interaction partner [29], was able to block binding of serum vitronectin to late apoptotic cells in a dose dependent manner (Fig. 3D). A possible explanation of this observation could be that heparin binding prevents recognition of the vitronectin molecule by the antibody. Thus, we prepared biotinylated red blood cells with bound biotinylated vitronectin using streptavidin as a bridge and examined the ability of anti-vitronectin antibody to recognize vitronectin in the presence of heparin. In this assay, heparin had no effect on the signal intensity (Fig. 3E), supporting the conclusion that heparin blocks vitronectin binding to the late apoptotic cells.

### 4. Vitronectin binds inside the damaged cells

**Figure 4:**
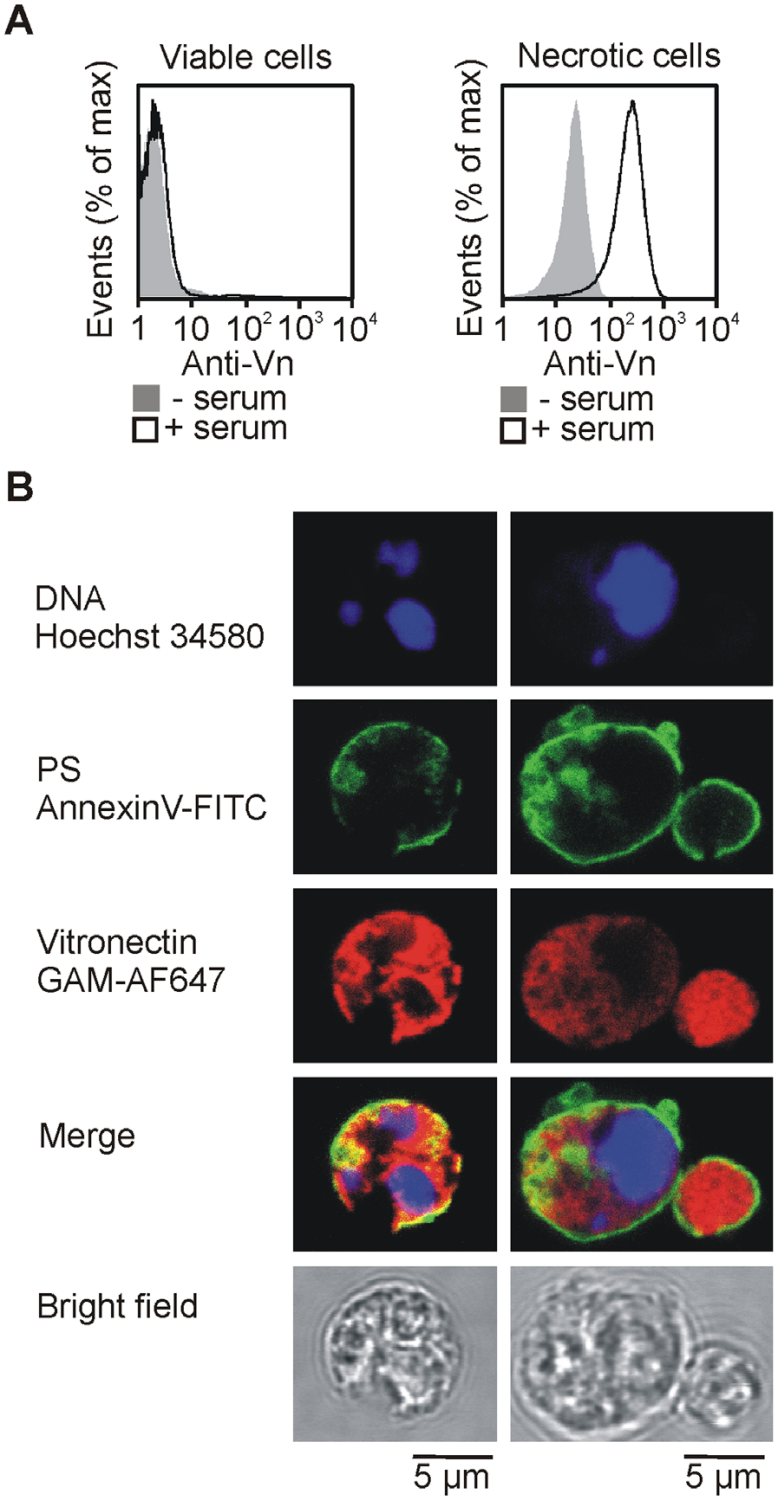
Vitronectin binds inside damaged cells. (A) Viable and heat-induced necrotic cells were incubated in the presence (black line) or absence (grey filled histogram) of human serum for 1 hour and stained with anti-vitronectin and GAM-Alexa Fluor 488 antibodies for flow cytometry analysis. One representative experiment of 3 is shown. (B) Apoptosis was induced in Jurkat cells by TRAIL in serum-free medium. After 72 hours, cells were incubated in 50% human serum for 1 hour. The cells were stained with anti-vitronectin and GAM-Alexa Fluor 647 antibodies followed by Annexin-V-FITC and Hoechst 34580. The cells were analyzed by confocal microscopy. Two representative cells (including a DNA-free apoptotic body) are shown. AF647, Alexa Fluor 647.

Late apoptotic cells share many features with necrotic cells (e.g. damaged plasma membrane). We tested the ability of serum vitronectin to bind Jurkat cells after heat-induced necrosis. Necrotic, but not viable, cells became positive for vitronectin after incubation in human serum (Fig. 4A).

We employed confocal microscopy to see whether vitronectin binds to the surface or inside the late apoptotic cells. Anti-vitronectin staining combined with Annexin-V membrane staining revealed clear surface localization of Annexin-V contrasting with diffuse intracellular staining for vitronectin in the late apoptotic cells and apoptotic bodies (Fig. 4B). As the cells were not fixed or permeabilized prior to the staining, the intracellular vitronectin signal implied that the late apoptotic cell membranes were permeable for vitronectin as well as for the antibodies. Regions of apoptotic fragmented nuclei stained by Hoechst 34508 were devoid of vitronectin indicating that vitronectin bound to cytoplasmic structures inside the late apoptotic cells (Fig. 4B). The vitronectin staining produced no signal when the pre-incubation in human serum was omitted; thus confirming the signal specificity (not shown). Additionally, microscopy also supported our flow cytometric data (Fig. 1B) demonstrating that vitronectin-positive cells contain only small amounts of DNA (Fig. S3).

### 5. Intermediate filaments do not mediate vitronectin binding

**Figure 5:**
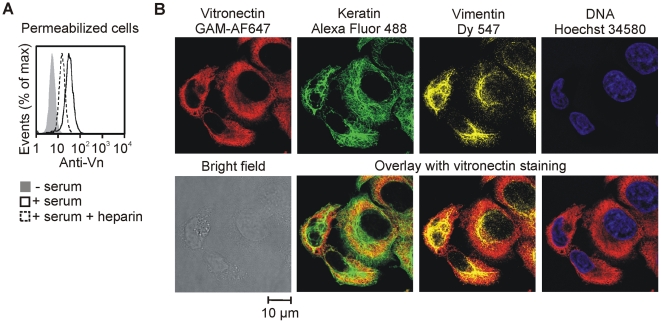
Vitronectin-binding cytoplasmic components are not intermediate filaments. (A) HeLa cells were harvested by trypsinization, fixed, and permeabilized. The cells were incubated in 50% human serum with (dashed line) or without heparin (50 IU/ml) (solid line) at 37°C for 1 hour. Afterwards, the cells were stained with anti-vitronectin and GAM-Alexa Fluor 647 antibodies and analyzed by flow cytometry. Cells not incubated in human serum served as negative controls (grey filled histogram). (B) HeLa cells cultivated on microscope cover slips were fixed and permeabilized. The cells were incubated in 50% human serum, stained with anti-vitronectin and GAM-Alexa Fluor 647 antibodies, followed by anti-pan-cytokeratin-Alexa Fluor 488 and anti-vimentin-Dy 547 antibodies in 20% mouse serum and Hoechst 34580 staining.

Since we detected vitronectin inside the ruptured apoptotic and necrotic cells, we hypothesized that the vitronectin binding partner might be present inside intact viable cells, but it is inaccessible to vitronectin due to the barrier represented by the plasma membrane. We tested the hypothesis using viable HeLa cells after detergent permeabilization. As expected, fixed and permeabilized HeLa cells interacted with human serum vitronectin in a heparin sensitive manner (Fig. 5A).

As vitronectin was shown to interact with vimentin and cytokeratins in vitro [30], [31], intermediate filament proteins seemed to be good candidates for vitronectin binding. However, confocal microscopy showed a diffuse cytoplasmic pattern of vitronectin staining in permeabilized HeLa cells after incubation in human serum. Moreover, vitronectin did not co-localize with vimentin or cytokeratins (Fig. 5B) indicating that vitronectin interacts with a cytoplasmic structure different from intermediate filament proteins.

### 6. Vitronectin binds late apoptotic/necrotic cells in vivo

**Figure 6:**
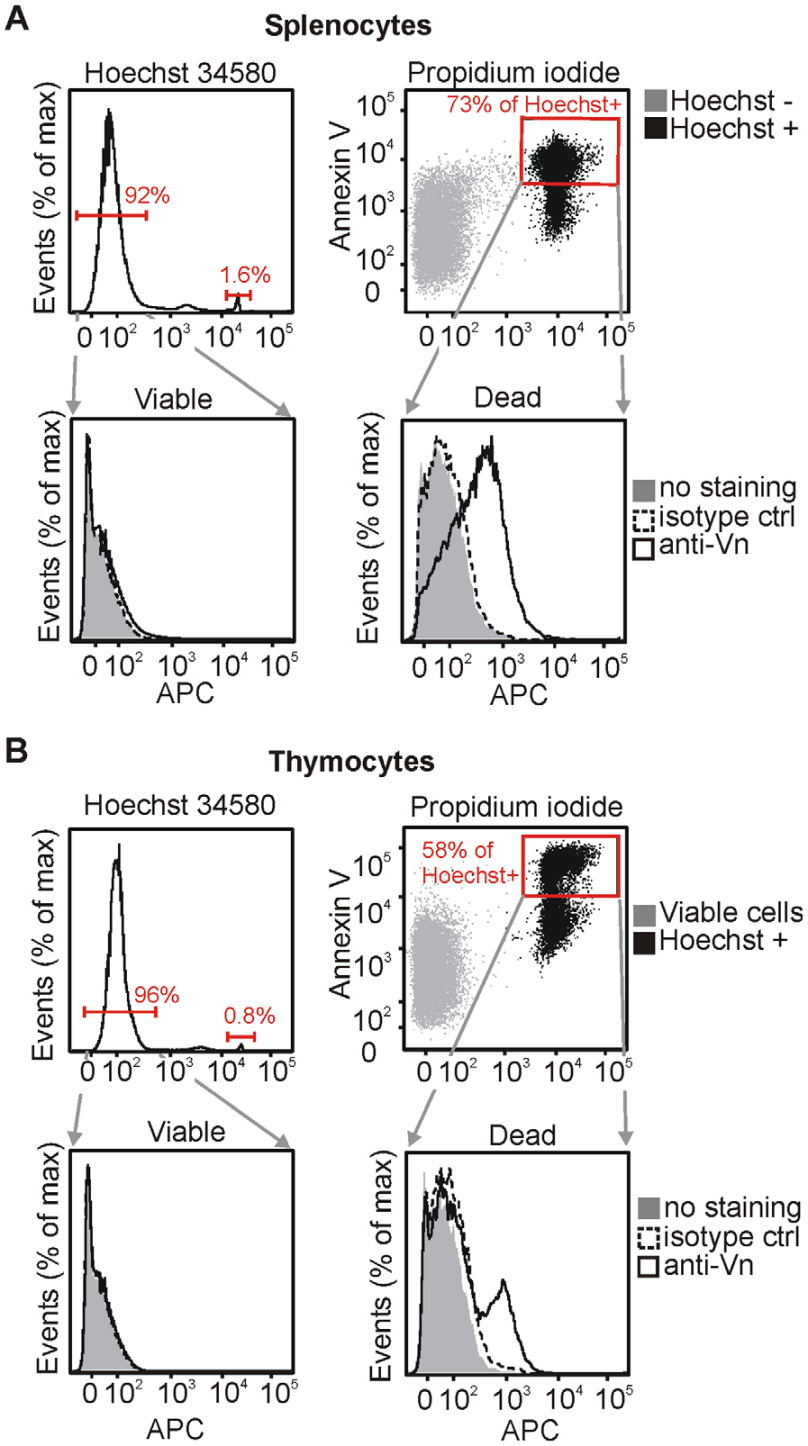
Vitronectin binds to dead cells in vivo. Single cell suspensions prepared from murine spleen (A) or thymus (B) were stained with antibody to mouse vitronectin or isotype matched control antibody, followed by secondary goat anti-rat-APC antibody, or left unstained. Simultaneously, staining with Hoechst 34580, PI, and Annexin-V-FITC was performed prior to flow cytometry analysis. Hoechst 34580 negative cells, that were essentially Annexin-V-FITC and PI negative, were considered as viable cells (upper panels). Hoechst 34580/Annexin-V-FITC/PI-triple positive cells were identified as dead cells (late apoptotic or necrotic) (upper right panels). APC-specific signal coming from staining with anti-vitronectin and isotype matched control antibodies on the viable and dead cells is shown (lower panels). One representative experiment of 3 is shown.

Experiments performed on cell cultures showed that serum vitronectin bound to the late apoptotic and necrotic cells in vitro. To elucidate whether vitronectin binds to the dead cells in vivo, we analyzed murine splenocytes and thymocytes in a four-colour flow cytometry assay. The cells were stained simultaneously with Hoechst 34580, PI, and Annexin-V for identification of late apoptotic/necrotic cells and with a rat antibody to mouse vitronectin. The splenocyte cell suspension contained about 1–2% of Hoechst 34580/PI/Annexin-V-triple positive necrotic/late apoptotic cells. Majority of these cells were positive for vitronectin, in contrast to essentially vitronectin-negative viable splenocytes (Fig. 6A). Thymus contained about 0.5% of late apoptotic/necrotic cells. About one quarter of these cells in thymus were positive for vitronectin whereas viable thymocytes were not stained with the anti-vitronectin antibody at all (Fig. 6B). The staining procedure was performed in the presence of 20% goat serum, which was not recognized by the antibody to mouse vitronectin (not shown). These results support the conclusion that vitronectin binds the late apoptotic/necrotic cells also in vivo.

### 7. Vitronectin as a tool for monitoring of cell death

As demonstrated above, vitronectin binds to cells with ruptured membrane in vitro and in vivo. When cells die in the presence of serum, the antibody recognizing vitronectin of the respective species can be used for detection of the terminal cell death phase (Fig. 1C, Fig. S2). If the cells are cultivated in the absence of vitronectin, a short incubation in the presence of serum or purified vitronectin must be included prior to the staining procedure (e.g. Fig 3A–C). Based on these results we developed an easy two-step cytofluorometric method for evaluation of the cell membrane integrity that works in all conditions regardless of the cultivation medium.

**Figure 7:**
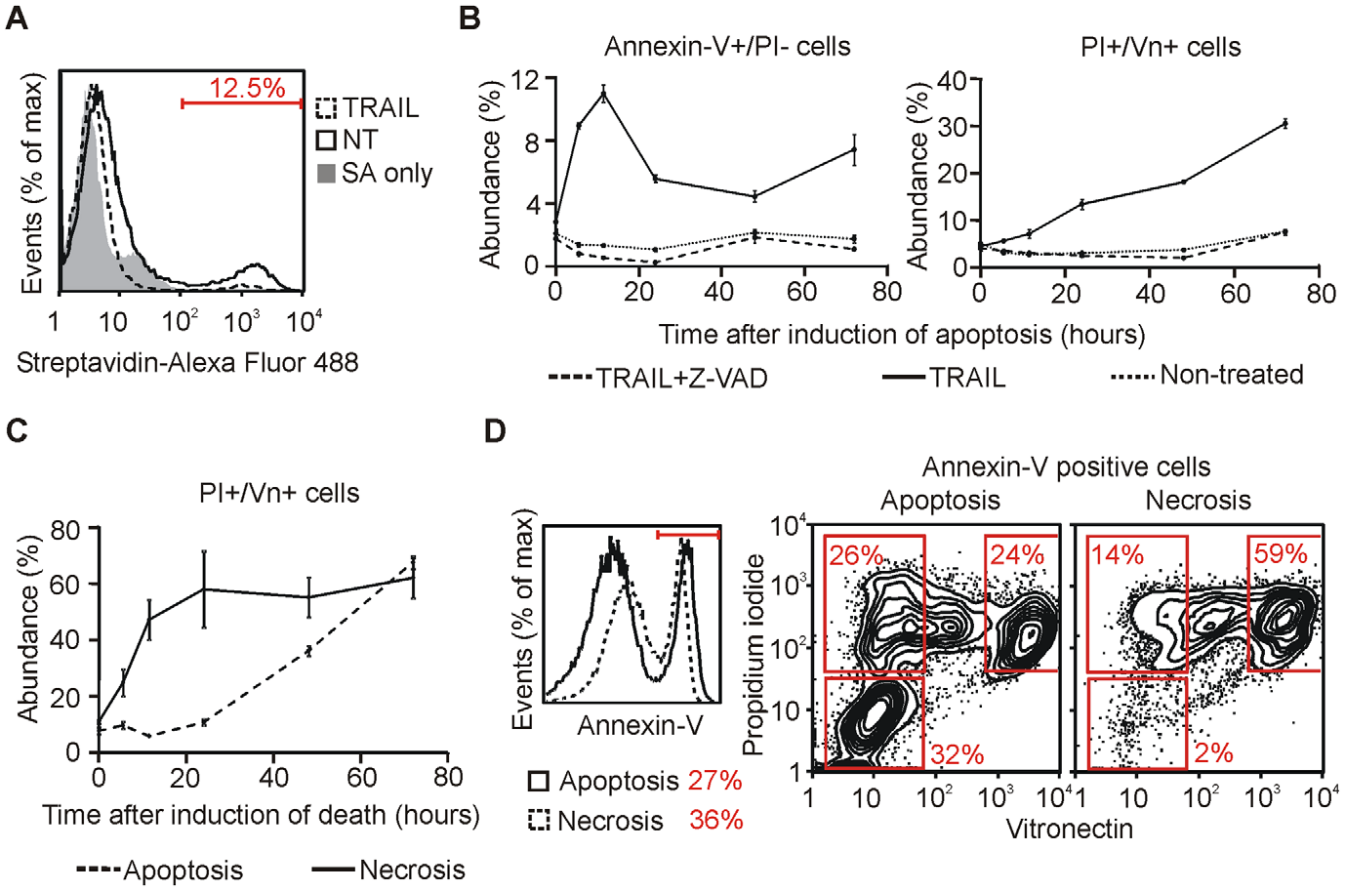
Vitronectin can be used for monitoring of cell death progression. (A) Apoptosis was induced in the Jurkat cells with TRAIL for 24 hours in FBS-containing medium. The cells were harvested and incubated with biotinylated vitronectin on ice for 30 minutes followed by staining with streptavidin-Alexa Fluor 488 and analyzed by flow cytometry. Non-treated cells and cells stained only with streptavidin were used as controls. (B) Apoptosis was induced in the Jurkat cells with TRAIL in FBS-containing medium. At the indicated time points, the cells were harvested and incubated with biotinylated vitronectin on ice for 30 minutes followed by staining with streptavidin-Alexa Fluor 488, propidium iodide, and Annexin-V-Dy647 and analyzed by flow cytometry. Non-treated cells and cells treated with TRAIL in combination with apoptosis inhibitor Z-VAD-FMK served as controls. Mean ± SD, n = 3. (C) Jurkat cells were subjected to apoptosis (TRAIL) or necrosis (hydrogen peroxide in combination with Z-VAD-FMK) induction in serum-free medium. At the indicated time points, the cells were harvested and incubated with biotinylated vitronectin on ice for 30 minutes followed by staining with streptavidin-Alexa Fluor 488 and flow cytometry analysis. Mean ± SD, n = 3. (D) Apoptosis (TRAIL) or necrosis (hydrogen peroxide in combination with Z-VAD-FMK) was induced in Jurkat cells in serum-free medium. After 18 hours, the cells were harvested and incubated with biotinylated vitronectin on ice for 30 minutes followed by staining with streptavidin-Alexa Fluor 488, propidium iodide, and Annexin-V-Dy647 and analyzed by flow cytometry. The Annexin-V-positive cells were gated (left panel) and propidium iodide vs. vitronectin staining was analyzed. Annexin-V^+^/PI^−^/Vn^−^, Annexin-V^+^/PI^+^/Vn^+^, Annexin-V^+^/PI^+^/Vn^high^ cells were gated (right panel). One representative experiment of 2 is shown.

We induced apoptosis of Jurkat cells with TRAIL in FBS-containing medium for 24 hours. The cells were stained with biotinylated vitronectin which was detected with streptavidin conjugated with Alexa Fluor 488 dye followed by flow cytometry. The number of vitronectin-positive cells increased after the apoptosis induction, showing that the method is compatible with cultivation in the presence of serum (Fig. 7A). Subsequently, we used this method for monitoring of cell cultures after apoptosis induction over time (Fig. 7B). The percentage of vitronectin-stained cells increased as the apoptosis progressed. No early apoptotic cells (AnnexinV^+^/PI^−^) nor vitronectin-stained cells appeared when the TRAIL-induced apoptosis was blocked with pan-caspase inhibitor Z-VAD, confirming that the vitronectin-positive cells were apoptotic.

As shown above, heat-induced necrotic cells become vitronectin-positive (Fig. 4A). However, necrosis can be induced by a milder stimulus that does not destroy cells immediately. Thus, we induced necrosis with hydrogen peroxide in combination with Z-VAD (to prevent possible apoptosis) and compared the cell death progression with TRAIL-induced apoptosis under serum-free conditions. Interestingly, the percentage of vitronectin-stained cells increased much more rapidly in the necrotic culture than in the apoptotic one (Fig. 7C). A triple staining with Annexin-V-Dy647, propidium iodide, and vitronectin showed that the character of the Annexin-V-positive cells differs substantially between the apoptotic and necrotic cells 18 hours after the particular cell death commitment (Fig. 7D). Annexin-V-positive apoptotic cells included comparable amounts of early apoptotic (PI^−^/Vn^−^), late apoptotic (PI^+^/Vn^−^), and ‘very late’ apoptotic (PI^+^/Vn^high^) cells. In contrast, Annexin-V-positive necrotic cells were mostly vitronectin highly positive and contained less PI^+^/Vn^−^ and only very few PI^−^/Vn^−^ cells. These results suggest that the progression of cell death resulting in complete membrane permeabilization is much faster in necrosis than in apoptosis.

## Discussion

2E12 monoclonal antibody was reported to stain a subset of late apoptotic cells cultured in vitro [5]–[6]. We identified the antigen recognized by 2E12 antibody as a bovine serum protein vitronectin that binds to apoptotic cells of various origin. Using a human T-cell line Jurkat and human vitronectin, we found that purified vitronectin binds late apoptotic cells in a dose-dependent manner. This binding could be inhibited by heparin. Confocal microscopy revealed that vitronectin binds inside the cells with severely damaged membrane. Moreover, vitronectin was detected in dead cells in mouse spleen and thymus suggesting that vitronectin binds necrotic cells and cells at the terminal stage of apoptosis in vivo.

Flow cytometry has been widely used to assess viability, apoptosis, and/or necrosis on a single cell basis [32]. A common method for detection of apoptotic cells is based on staining with Annexin-V and a DNA-binding dye that does not penetrate intact cell membrane (e.g. PI). Annexin-V-positive and PI-negative cells are considered as “early apoptotic” while the double positive cells are classified as “late apoptotic” or “necrotic”. However, it has been documented that the loss of the membrane integrity is a gradual process. First, the membrane of a late apoptotic cell becomes permeable for small molecules (PI) and subsequently opens also for macromolecules [4], [33]–[35]. The latter phase of membrane damage is usually monitored as a leakage of lactate dehydrogenase or other intracellular proteins using enzymatic assays or immunoblotting. Our observations suggest that a simple flow cytometry approach based on vitronectin staining can be used to distinguish between the two subsets of late apoptotic cells on a single cell level. Such classification of the apoptotic stages could be biologically important. In contrast to vitronectin-positive cells, the PI^+^/Vn^−^ cells can still retain macromolecules that could induce immunogenic or inflammatory response in case of their escape [4]. We used this method to reveal that necrosis induced by oxidative stress (hydrogen peroxide) or heating leads to complete membrane permeabilization much faster than apoptosis. Apoptotic cells are apparently able to keep their membranes non-permeable for proteins and other macromolecules for some time even after they become permeable for small charged molecules like propidium iodide. Thus, they can extend the time needed for their clearance before the potential auto-antigens are released into the body. In contrast, necrotic cells become permeable for proteins either immediately or shortly after the onset of necrosis.

Vitronectin is an abundant plasma glycoprotein and is a part of the extracellular matrix [7], [8], [14], [15]. While the plasma vitronectin is produced mainly by the liver, the origin of the tissue vitronectin is less clear [8]. Most probably the tissue vitronectin originates mainly from the plasma vitronectin that is translocated across endothelium via active transcytosis [8], [36], [37]. Alternatively, vitronectin could be produced locally in the tissue. This is supported by the detection of vitronectin mRNA in several tissues albeit at much lower level than in the liver [8]. Vitronectin is substantially enriched in the sites of inflamed, injured, necrotic, and cancer tissues including cirrhotic liver, atherosclerotic plaques, injured skin, Alzheimer plaques, myocardial infarction, and colorectal carcinoma [8]–[12], [15], [25], [29], [31], [38]–[41]. Interestingly, atherosclerosis and Alzheimer disease are also associated with defects in apoptotic cell clearance [3].

Little is known about the mechanisms regulating the delivery of vitronectin specifically into the sites of injured tissue. Two possible explanations were suggested: (1) vitronectin leakage from capillaries at the sites of injury and (2) an interaction between vitronectin and a component specifically present in the stressed tissue [8]. Here we show that vitronectin binds to necrotic and late apoptotic cells in cell culture and in vivo. This supports the model that vitronectin incorporation into the sites of injured, stressed, and, possibly, also cancer tissue is at least partially dependent on the interactions with a cytoplasmic component exposed after tissue injury.

Vitronectin was shown to bind to in vitro prepared keratin bodies and to keratinocyte derived Civatte bodies in patients suffering from lichen ruber planus [30], [42]. Moreover, vitronectin was shown to interact with purified vimentin and with vimentin exposed on the surface of activated platelets [31], [43]. However, we show here that the intracellular vitronectin-interacting structure is diffusely distributed in the cytoplasm of viable cells and does not co-localize with vimentin or cytokeratine filaments. Thus, the binding of vitronectin to necrotic and late apoptotic cells is apparently mediated mainly by a so far unidentified component different from the intermediate cytoskeletal proteins.

Among numerous vitronectin binding receptors, α_V_β_3_, α_V_β_5_ integrins and the urokinase receptor are involved in the recognition and engulfment of apoptotic cells by phagocytes [2], [44], [45]. However, other ligands of these receptors, including milk fat globule-EGF factor 8 and thrombospondin, were found to mediate phagocytosis of apoptotic cells [3], [46], [47] while there was only indirect evidence for possible vitronectin involvement [48]–[50]. Despite numerous attempts, we did not observe any effects of vitronectin opsonization on phagocytosis of the late apoptotic cells by human monocytes, macrophages, or dendritic cells (not shown). Accordingly, depletion of vitronectin from bovine serum also did not induce any changes in phagocytosis of the late apoptotic cells (not shown).

In conclusion, our observations that vitronectin binding does not serve as an opsonization step to facilitate phagocytosis indicates that this interaction may be rather relevant for other biological roles of vitronectin in regeneration processes in injured tissues (i.e. tissue remodeling, cell survival, and inflammation) that were documented in several previously published studies [16]–[18], [21], [22].

## Supporting Information

Figure S1 **2E12 antibody stains a subset of hypodiploid cells of various species.** Cell lines from different species were stained with Hoechst 34580 and 2E12 antibody for flow cytometry analysis. Human cell lines: JCaM2.5, Ramos, HL-60; chicken cell line: DT40; rabbit cell line: 240E1; mouse cell lines: SP2/0, P815.
(TIF)

Figure S2 **Human vitronectin binds to cells in the late phases of apoptosis.** Apoptosis was induced in Jurkat cells by campthotecin. The cells were incubated in RPMI/10% human AB serum. At indicated time points, cells were stained with Annexin-V-FITC, propidium, and antibody to human vitronectin+GAM-Alexa Fluor 647 and analyzed by flow cytometry. Mean ± SD, n = 3.
(TIF)

Figure S3 **Vitronectin positive late apoptotic cells exhibit low DNA content.** Late apoptotic Jurkat cells were incubated in human serum and stained with antibody to human vitronectin followed by Alexa Fluor 647 conjugated secondary antibody (red colour) and with DNA dye Hoechst 34580 (blue colour). The white arrows point to vitronectin^low^/DNA^high^ cells, green arrows point to vitronectin^high^/DNA^low^ cells.
(TIF)
