# Supplementary figures and images for: Interaction of Late Apoptotic and Necrotic Cells with Vitronectin
Source: PLoS One. 2011 May 4;6(5):e19243. doi: 10.1371/journal.pone.0019243 (PMC3087723; doi:10.1371/journal.pone.0019243)

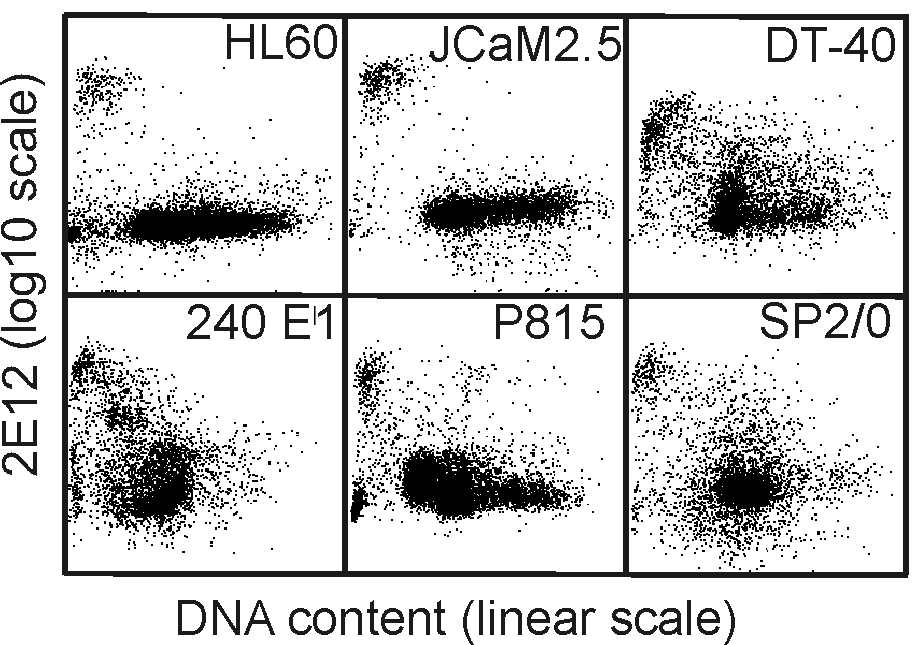

Supplement: Figure S1 — 2E12 antibody stains a subset of hypodiploid cells of various species. Cell lines from different species were stained with Hoechst 34580 and 2E12 antibody for flow cytometry analysis. Human cell lines: JCaM2.5, Ramos, HL-60; chicken cell line: DT40; rabbit cell line: 240E1; mouse cell lines: SP2/0, P815. (TIF) [file pone.0019243.s001.tif]

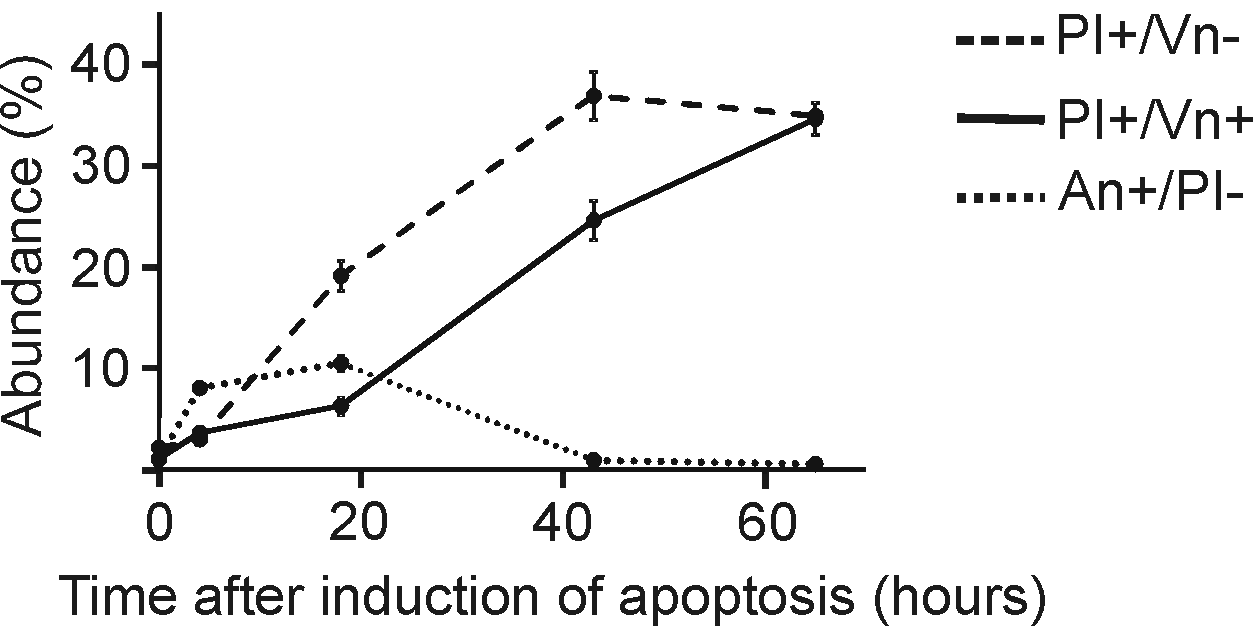

Supplement: Figure S2 — Human vitronectin binds to cells in the late phases of apoptosis. Apoptosis was induced in Jurkat cells by campthotecin. The cells were incubated in RPMI/10% human AB serum. At indicated time points, cells were stained with Annexin-V-FITC, propidium, and antibody to human vitronectin+GAM-Alexa Fluor 647 and analyzed by flow cytometry. Mean ± SD, n = 3. (TIF) [file pone.0019243.s002.tif]

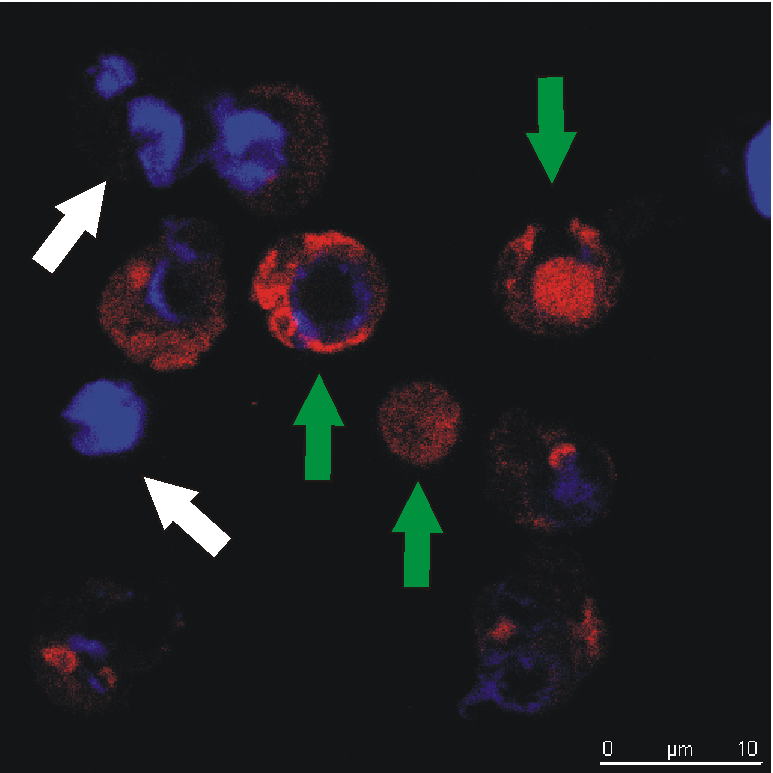

Supplement: Figure S3 — Vitronectin positive late apoptotic cells exhibit low DNA content. Late apoptotic Jurkat cells were incubated in human serum and stained with antibody to human vitronectin followed by Alexa Fluor 647 conjugated secondary antibody (red colour) and with DNA dye Hoechst 34580 (blue colour). The white arrows point to vitronectinlow/DNAhigh cells, green arrows point to vitronectinhigh/DNAlow cells. (TIF) [file pone.0019243.s003.tif]
